# Development of TaqMan-based qPCR method for detection of caprine arthritis–encephalitis virus (CAEV) infection

**DOI:** 10.1007/s00705-013-1728-1

**Published:** 2013-05-14

**Authors:** Yi Li, Fengjuan Zhou, Xia Li, Jianhua Wang, Xiangping Zhao, Jinhai Huang

**Affiliations:** 1 School of Chemical Engineering and Technology, Tianjin University, Tianjin 300072, China. No. 92, Weijin road, Nankai District, Tianjin, 300072 China; 2 Tianjin Animal, Plant and Food Inspection and Quarantine Inspection, Tianjin, 300000 China

## Abstract

A specific and sensitive two-step TaqMan real-time PCR has been developed for rapid diagnosis of caprine arthritis-encephalitis virus (CAEV) infection by using a set of specific primers and a TaqMan probe targeting a highly conserved region within the gene encoding the viral capsid protein (CA). The assay successfully detected CAEV proviral DNA in total DNA extracts originating from cell culture, whole blood samples and isolated PBMCs, with a lower detection limit of 10^2^ copies and a linear dynamic range of 10^5^ to 10^10^ copies/ml. There was no cross-reaction with other animal viruses (e.g., goat pox virus, bovine leukemia virus, bovine mucosal disease virus, swine influenza virus and Nipah virus). When applied in parallel with serological AGID and conventional PCR for detection of CAEV in field samples, this assay exhibited a higher sensitivity than these traditional methods, and 7.8 % of the 308 specimens collected in the Shanxi and Tianjin regions of China from 1993 to 2011 were found to be positive. Thus, the TaqMan qPCR assay provides a fast, specific and sensitive means for detecting CAEV proviral DNA in goat specimens and should be useful for large-scale detection in eradication programs and epidemiological studies.

## Introduction

Caprine arthritis-encephalitis virus (CAEV) is a member of the genus *Lentivirus*, family *Retroviridae* [4] and induces persistent and progressive degenerative inflammatory disease in infected goats [20]. Although most infected goats remain asymptomatic, they are lifelong carriers and keep shedding the virus to the environment, leading to infection of naïve goats. Nevertheless, after prolonged incubation, a substantial population of CAEV-infected goats develop clinical signs primarily characterized by leukoencephalomyelitis in kids [25] and chronic polyarthritis and indurative mastitis in adults [8]. CAEV is mainly macrophage-tropic; expression of the viral genome depends on the maturation state of the cells, and viral transcripts are produced only when the cells mature into macrophages [9, 21]. Epidemiological evidence indicates that the virus is transmitted from infected does to their offspring through the consumption of virus-infected colostrum and milk [29] or through prolonged close contact with infected adult animals [2].

CAEV infection is one of the most destructive and economically important viral diseases of the goat industry and is spread throughout many countries of the world, including the United States (31 %) [30], Norway (86 %) [22], Great Britain (54.5 %) [32], Switzerland (26.9 %) [3], Spain (20.6 %) [31], Poland (12.1 %) [15], Italy (6.58 %) [11], Japan (63.3 %) [14], Mexico (56.8 %) [33], Brazil (35 %) [18], Jordan (23.2 %) [1], Korea (2.73 %) [23] and China (0.2 %–30 %) [26]. Overall, the live-animal trade and exportation of goats play a major role in CAEV dissemination across large geographical regions [24]. Economic losses attributed to CAEV infection are considerably adverse in countries with intensive animal husbandry, with 5 %–10 % goats reported to be culled annually due to arthritis, and the decrease in milk production in infected does was estimated to be 10 %–15 % in Switzerland [24]. The differences in the content of protein (3.35 % vs. 3.40 %), fat (3.54 % vs. 3.69 %), and lactose (4.25 % vs. 4.30 %) between seropositive and seronegative milk are significant [13]. In udder halves with intramammary infection, milk SCC (somatic cell count) was significantly increased [17].

Currently, there are no effective drugs or vaccines available for treatment or prevention of CAEV infection. Therefore, immediate and accurate diagnosis is of particular importance for identifying and culling CAEV-positive animals from the rest of the herd to reduce economic losses [34]. Routine laboratory diagnosis of CAEV infection is mainly based on serological assays [19]. An agar gel immunodiffusion (AGID) test, an assay that is based on the CAEV serology, is recommended by the Office International des Epizooties (OIE). Enzyme-linked immunosorbent assay (ELISA), which uses recombinant capsid (CA) or TM envelope protein subunits as antigen [6, 27, 35], has proven more sensitive than AGID; however, the antigenic heterogeneity of CA and TM [10] may result in a lack of sensitivity, if the animal was infected with a lentivirus genotype different from that employed in the assay [28]. Additionally, preparation of antigen is expensive, time-consuming and unpractical for routine diagnosis [5].

During our routine surveillance, we found that 4 out of 34 AGID-seronegative animals were PCR positive, suggesting that the classical management practice (AGID) recommended for CAEV control is insufficient [19]. PCR-based diagnostic techniques vary according to their targets, such as reverse transcription PCR for the detection of viral RNA [16], semi-nested PCR [7] or loop-mediated isothermal amplification (LAMP) of proviral DNA [12], and real-time PCR detection of the CAEV env gene. The real-time PCR gave earlier positive detection results (15 days postinfection) than serological methods(ELISA and AGID, about 40-60 days postinfection) [2]. An early/fast laboratory diagnosis for CAEV infection can be very useful for effective prophylactic action, and PCR is a useful tool for decreasing the risk of breeding AGID-false-negative animals [19]. The aim of this study was to develop a TaqMan-based qPCR method to detect and quantify CAEV DNA in infected goat tissues by targeting a highly conserved region encoding the viral capsid protein (CA).

## Materials and methods

### Cells and viruses

GSM (goat synovial membrane) cells were maintained in DMEM supplemented with 10 % FBS at 37 °C with 5 % CO_2_. The cells were infected with the CAEV-Shanxi strain and monitored daily for cytopathic effect (CPE), as indicated by multinucleated cells and refractile stellate cells. When CPE reached to 50 to 80 %, cells were harvested and stored at −80 °C until used for nucleic acid extraction. DNA extracts of goat pox virus and bovine leukemia virus from cell culture and cDNA of swine influenza virus, bovine mucosal disease virus and Nipah virus were provided by the Tianjin Entry-Exit Inspection and Quarantine Bureau.

### Nucleic acid extraction

Total DNA from PBMCs and infected cell cultures was extracted using a TIANamp Genomic DNA Kit (Tiangen Biotech Inc., Beijing, China), following the manufacturer’s instructions. The DNA was quantified by spectrophotometry (Nano Drop 1000 Spectrophotometer, Thermo Fisher Scientific Inc.) and stored at −20 °C until TaqMan qPCR was performed. CAEV RNA from a 250-μL sample containing 10^5^ TCID_50_ of CAEV Shannxi strain culture was extracted using a QIAamp Viral RNA Mini Kit, and cDNA synthesis was performed using reverse transcriptase (QIAGEN, Beijing, China).

### Conditions for TaqMan PCR and PCR

For TaqMan qPCR, primers and a probe corresponding to a highly conserved region in the *CA* gene of the CAEV genome were designed using Beacon Designer 7.0 software (http://www.premierbiosoft.com) and synthesized by Invitrogen (Beijing) (Table 1). The probe was labeled with reporter and quencher dye (FAM, TAMRA) at its 5′ and 3′ end, respectively.

**Table 1:** TaqMan qPCR primers and probe

Target gene (bp)	Primer/probe	Sequence (5′–3′)	Position	Amplicon size (bp)
CA	CAF	AGGTGGAGAAGAAATAATCC	1120-1139	149
	CAP	FAM-TGTCTTGCCTGATCCATGTTAGC-TAMRA	1238-1216	
	CAR	AAGGCTATTATTACCCATTG	1268-1249	

DNA extracted from GSM (goat synovial membrane) cells infected with the CAEV-Shanxi strain was used as a positive control, while those from seronegative goats or mock-infected GSM cells or PBMCs of CAEV-negative goats served as negative control. The TaqMan qPCR was carried out using Platinum^®^ Quantitative PCR SuperMix-UDG with ROX (Invitrogen, Beijing, China) with a final concentration of 0.125 μM primers and 0.2 μM TaqMan probe (Table 1). The reaction mixture also included 1.0 μL plasmid DNA (approximately 0.038 μg), 25 μL of 2 × reaction mix and DNase-free water in a final volume of 50 μL. The TaqMan qPCR was performed on an ABI Prism^®^ 7900HT instrument (Applied Biosystems, USA). The cycling conditions included an initial UDG (uracil-DNA glycosylase) incubation step at 50 °C for 2 min, followed by denaturation at 95 °C for 2 min to activate the Platinum^®^ Taq DNA polymerase. Amplification was then performed using 40 cycles of denaturation at 95 °C for 15 s and annealing and extension at 54 °C for 30 s. Fluorescent signals were obtained once per cycle upon completion of the extension step at the wavelengths corresponding to FAM fluorescence (520 nm). Data acquisition and analysis were performed using the ABI Prism^®^ 7900HT data analysis SDS software (Table 2).

**Table 2:** Number of positive and negative samples detected by TaqMan qPCR, PCR assay and AGID

Group	No. of Samples	No. of positive samples (%)			Coincidence (%)
		TaqMan qPCR	Conventional PCR	AGID	
A	48	17 (35.4)	16 (33.3)	15 (31.3)	46 (95.8)
B	20	7 (35)	7 (35)	6 (30)	19 (95)
C	240	0 (0)	0 (0)	0 (0)	240 (100)
Total	308	24 (7.8)	23 (7.5)	21 (6.8)	305 (99.1)

The primers CAF and CAR for TaqMan qPCR were also used for regular PCR. The PCR reaction mixture included 1 μL of plasmid DNA (approximately 0.038 μg), 0.25 μM CAF and 0.25 μM CAR, 0.2 μM dNTP mixture, 5 μL 10 × PCR buffer, 2.5 U of Platinum^®^ Taq DNA polymerase (Invitrogen Beijing, China), and DNase-free water in a final volume of 50 μL. The PCR amplification parameters included an activation step at 95 °C for 10 min, 35 cycles of amplification (94 °C for 30 s, 55 °C for 30 s, and 72 °C for 30 s) and a final extension at 72 °C for 5 min. PCR products were electrophoresed in a 1.5 % agarose gel, stained with ethidium bromide, and visualised under UV light.

### Sensitivity and specificity of the TaqMan qPCR and PCR

To generate a DNA standard curve, the gene encoding the CA capsid protein was cloned into the pGEM-T vector to generate the recombinant plasmid pGEM-T-CA. The concentration of the plasmid was determined by measuring OD absorbance at 260 nm, and the copy number was calculated by the following formula: plasmid copy (copies/μL) = [plasmid DNA concentration (g/μL) × 6.02 × 10^23^]/[length of DNA (bp) × 660]. The recombinant plasmid pGEM-T-CA was then serially diluted in tenfold steps, ranging from 10^10^ to 10^5^ copies/ml, and used to generate a standard curve for quantification.

CAEV mRNA was extracted from GSM cells infected with the CAEV Shanxi strain, using an RNeasy Mini Kit, QIAGEN) and then reverse transcribed with primer CAR. The sensitivity of TaqMan qPCR and regular PCR was subsequently determined using a series of tenfold-diluted cDNA as template. The specificity of the TaqMan qPCR and PCR assay was evaluated by cross-reaction tests using DNA or cDNA templates extracted from some other animal viruses, including swine influenza virus, goat pox virus, bovine leukemia virus, bovine mucosal disease virus and Nipah virus. All of those virus specimens were provided by the Tianjin Entry-Exit Inspection and Quarantine Bureau.

### Detection of CAEV in field samples

A total of 308 field samples were used to evaluate the sensitivity and specificity of the TaqMan qPCR in comparison to other traditional methods (Table 2). Since these samples were collected from three different herds with a history of CAEV infection in 1993, 2001 and 2010-2011, they therefore were divided into three groups (A, B and C). Group A consisted of 48 goats that originated from 8 flocks in Shanxi Province with a history of CAEV infection, group B samples were collected in 2001 from 20 goats from a mixed flock where sheep and goats were kept together in Tianjin City, and group C consisted of 240 goats that came from two flocks in Tianjin.

All blood samples were collected by jugular vein puncture. To isolate PBMCs, 10 ml of whole blood were collected in vacutainer tubes with EDTA, layered on an equal volume of Histopaque-1077 (Sigma-Aldrich, Germany), and then spun at 400×*g* for 30 min. The PBMC-containing layer was collected and washed twice, first with 10 ml and then with 5 ml isotonic phosphate-buffered saline solution, and used for extraction of genomic DNA. Twenty nanograms of total DNA was used for TaqMan qPCR and PCR detection.

## Results

### Sensitivity of the TaqMan qPCR and PCR assay

**Fig. 1:**
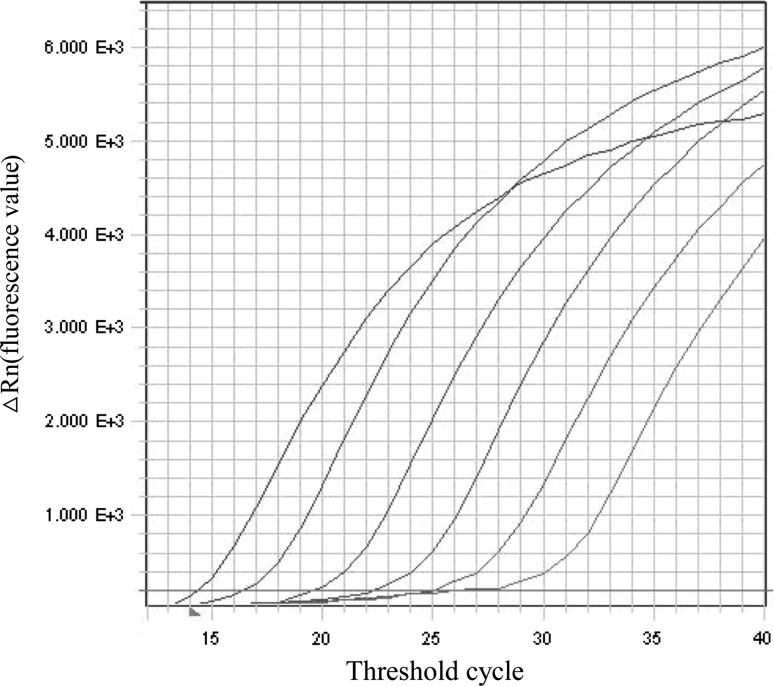
TaqMan qPCR amplification plots. Tenfold serial dilutions of the recombinant plasmid pGEM-T-CA (ranging from 10^10^ to10^5^ copies/mL) were prepared, and 1 μL of each serially diluted recombinant plasmid sample was used as a template in the assay

To determine the sensitivity of the TaqMan qPCR assay, the recombinant plasmid pGEM-T-CA (ranging from 10^10^ to10^5^ copies/mL) was prepared in tenfold serial dilutions, and 1 μL of each serially diluted recombinant plasmid sample was used as template. As expected, the threshold cycle (Ct) increased in inverse proportion to concentration of the recombinant plasmid standard (Fig. 1). The sample was judged as positive when the Ct value ranged from 10 to 35.

**Fig. 2:**
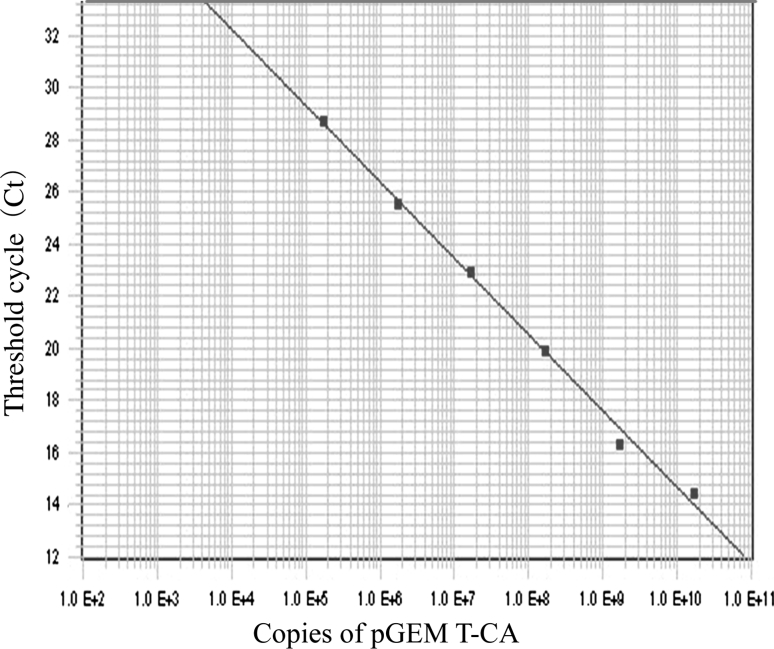
TaqMan qPCR standard quantification curve. A standard quantification curve (Ct values plotted versus the sample dilution) derived from the TaqMan qPCR amplification plots (in Fig. 1). The linear range of quantitation was 10^5^-10^10^ copies/mL plasmid/TaqMan qPCR. The correlation regression coefficient R^2^ is 0.99, and the detection limit is 10^2^ copies/μL plasmid (not shown in Fig. 2)

This TaqMan qPCR assay had a detection limit of 100 copies/μL plasmid. The wide linear range (10^5^-10^10^ copies/mL) is illustrated in Fig. 2. Standard curves showed a good correlation regression coefficient R^2^ of 0.99.

**Fig. 3:**
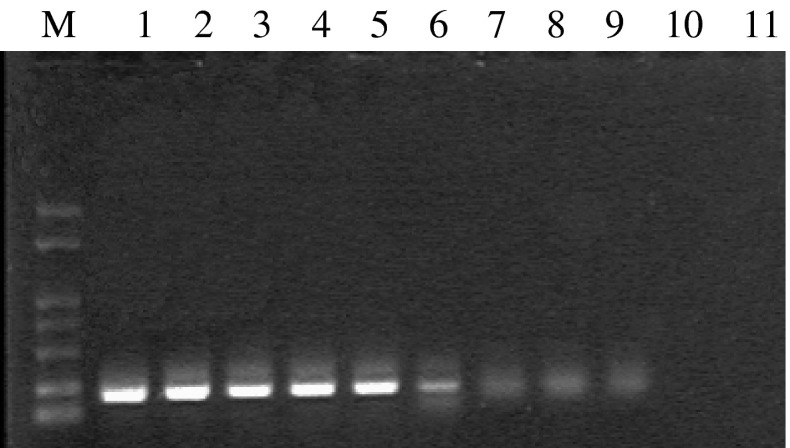
Sensitivity of the conventional PCR assay. Serial tenfold dilutions of the recombinant plasmid pGEM-T-CA were used to in the PCR assay. From CH1-CH10, the copies of plasmid decreased from 10^10^ to 10^1^ copies per μL. ddH_2_O was used as a negative control (CH11). Lane M, DNA Marker DM1000. The detection limit is 10^2^ copies per μL plasmid

The sensitivity of conventional PCR using CAF and CAR primer (Fig. 3) is lower or similar to that of TaqMan qPCR. The relationship between the sensitivity of TaqMan qPCR and the number of virus particles was evaluated using viral RNA extracted from CAEV-infected GSM cells, and about 0.001 TCID_50_ virus particles could still be detected.

### Specificity of TaqMan qPCR

**Fig. 4:**
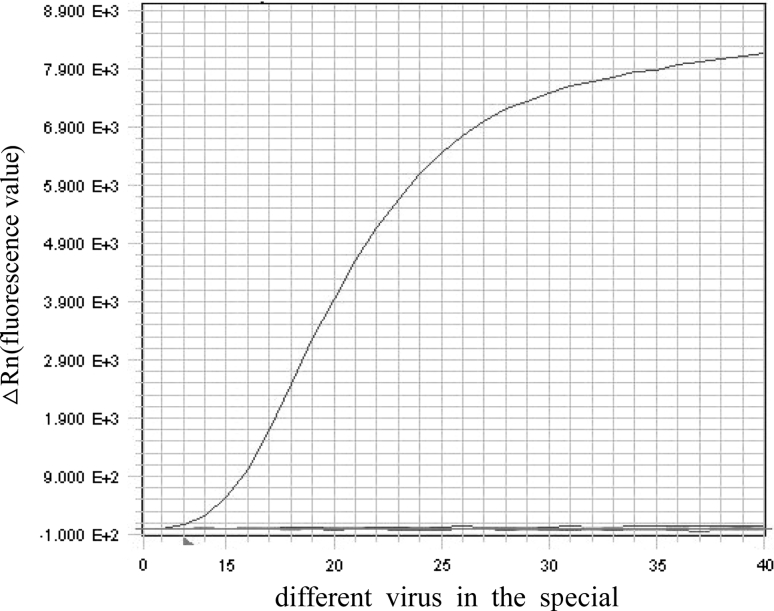
Specificity of the TaqMan qPCR assay. The amplification of the CAEV-positive control sample is apparent; other viruses including swine influenza virus, bovine leukemia virus, goat pox virus, bovine viral diarrhea-mucosal disease, and Nipah virus, as well as the CAEV-negative control sample and the blank control, show no amplification. The DNA concentration of each sample was adjusted to 20 ng/μl, and 1 μL was then added to a total volume of 50 μL reaction mixture. Only the positive CAEV samples showed amplification

There was no cross-amplification signal when other animal viruses such as swine influenza virus, goat pox virus, bovine leukemia virus, bovine mucosal disease virus and Nipah virus were tested (Fig. 4). The specificity of the TaqMan qPCR was compared with that of regular PCR and AGID, and positive amplification reactions occurred in all AGID-positive samples; however, TaqMan assays detected an additional three samples (two samples in group A and one sample from group B) that were missed by AGID. The newly developed TaqMan PCR based on the CA gene of CAEV had a similar sensitivity to that of the assay described by Brajon et al. in which the env gene of CAEV was detected by real-time PCR.

### TaqMan qPCR detection of CAEV in field samples

**Fig. 5:**
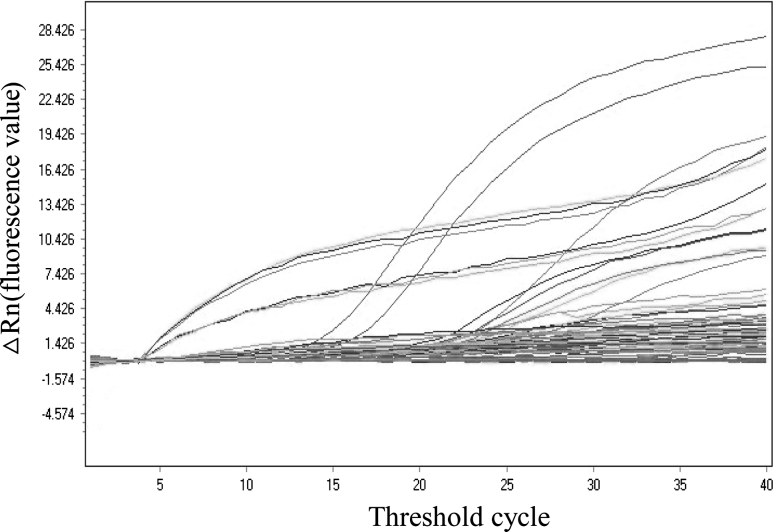
Detection of CAEV-positive and negative samples by TaqMan qPCR assay. Three hundred eight specimens containing CAEV were used to evaluate the efficiency of the TaqMan qPCR assay. CAEV-positive samples, CAEV-negative samples, and 45 specimens were tested simultaneously in a 96-well plate. Seven out of 45 samples were CAEV positive, and all of the positive samples detected were from herd A

To further evaluate its sensitivity, the TaqMan qPCR assay was applied in parallel with traditional serological AGID and conventional PCR to detect CAEV in 308 field samples (Fig. 5). In group A, TaqMan qPCR picked up one more sample than the conventional PCR assay, and 17 out of 48 samples from PBMC DNA preparations were CAEV positive. For groups A and B, all AGID-positive goats were also positive by TaqMan and regular PCR; however, TaqMan assays detected an additional three samples (two samples in group A and on in group B) that were missed by AGID. In the case of the remaining 20 samples collected in Tianjin in 2001 (group B), the TaqMan qPCR showed the same sensitivity as the conventional PCR. The coincidence of the three methods was 95.6 % for group A and B animal samples. For the samples from group C, all 240 specimens were negative by all three assays. No amplification signals were obtained with the negative controls or the GSM cell control. Overall, when all 308 clinical samples are considered, the TaqMan assay had good consistency and correlation with conventional PCR and AGID. The total CAEV-positive rate with TaqMan qPCR (7.8 %) was slightly higher than that obtained by conventional PCR (7.5 %) and AGID (6.82 %), although this difference was not statistically significant.

## Discussion

CAEV control has remained a big challenge for the goat industry, as prophylactic vaccinations do not induce antibodies that result in efficient viral clearance and provide protection against arthritis [11]. The control measures therefore rely heavily on accurate and reasonable laboratory diagnosis to identify and cull the CAEV-infected subjects so as to reduce economic losses [2].

PCR-based methods are now routinely used for laboratory diagnosis of pathogens, with acceptably high specificity. Based on regular PCR, we have developed a TaqMan qPCR assay for rapid CAEV diagnosis. This assay was simple to carry out and sensitive enough to detect viral DNA directly from PBMCs. Our results showed that the detection rate of TaqMan qPCR is higher than that of conventional PCR. Due to strain variation and the low viral load *in vivo*, the choice of the target region of the primers and probes can affect the efficiency of a PCR assay, and we therefore chose the region coding for viral capsid protein. This region is conserved in the CAEV genome, and it is obviously more suitable than other regions for the purpose of viral detection. Furthermore, the primers recognizing this region have high specificity and did not cross-amplify the sequences from swine influenza virus, goat poxvirus, bovine leukemia virus, bovine mucosal disease virus and Nipah virus.

As reported previously [2], real-time PCR can identify an infection much earlier (15 days postinfection) than serological methods (ELISA and AGID, 40-60 days postinfection). When applied to clinical diagnosis of CAEV, this method serves as a feasible and attractive method for large-scale screening, particularly at times of CAEV outbreaks. Rapid laboratory diagnosis of CAEV infections at an early stage of the disease can yield information relevant to goat industry management and help facilitate biosecurity protocols.

In Tianjin, the prevalence of CAEV-positive animals decreased during the years 2001 to 2010-2011. This is largely attributed to the measures for CAEV eradication that were taken in most farms of Tianjin, including serum antibody detection by AGID and viral DNA detection by PCR. Following diagnosis, all positive goats were slaughtered and removed, newborn kids were disinfected and separated from their does, and the kids were fed with milk or milk replacer rather than colostrum from CAEV-infected does. Other biosecurity practices were also implemented. In conclusion, the emphasis on identification CAEV infection is clearly evident, and the TaqMan qPCR method will be a sensitive, specific, and effective tool in CAEV control and eradication programs.

## References

[1] Al-Qudah K, Al-Majali AM (2006). Epidemiological studies on caprine arthritis-encephalitis virus infection in Jordan. Small Rumin Res.

[2] Brajon G, Mandas D (2012). Development and field testing of a real-time PCR assay for Caprine Arthritis–Encephalitis-virus (CAEV). Open Virol J.

[3] Brulisauer F, Vogt HR (2005). Risk factors for the infection of Swiss goat herds with small ruminant lentivirus: a case-control study. Vet Rec.

[4] Cork LC, Hadlow WJ (1974). Infectious leukoencephalomyelitis of young goats. J Infect Dis.

[5] Crespo Leon F, Gutierrez Diez F (2005). The translation into Spanish of the OIE manual of diagnostic tests and vaccines for terrestrial animals (mammals, birds and bees): problems, solutions and conclusions. Rev Sci Tech.

[6] de Andrés D, Klein D (2005). Diagnostic tests for small ruminant lentiviruses. Vet Microbiol.

[7] Eltahir YM, Dovas CI, et al (2006) Development of a semi-nested PCR using degenerate primers for the generic detection of small ruminant lentivirus proviral DNA. J virol met 135(2):240-246 10.1016/j.jviromet.2006.03.010 16650487

[8] Gjerset B, Jonassen CM (2007). Natural transmission and comparative analysis of small ruminant lentiviruses in the Norwegian sheep and goat populations. Virus Res.

[9] Gorrell MD, Brandon MR (1992). Ovine lentivirus is macrophagetropic and does not replicate productively in T lymphocytes. J Virol.

[10] Grego E, Profiti M (2002). Genetic heterogeneity of small ruminant lentiviruses involves immunodominant epitope of capsid antigen and affects sensitivity of single-strain-based immunoassay. Clin Diagn Lab Immunol.

[11] Gufler H, Gasteiner J (2007). Serological study of small ruminant lentivirus in goats in Italy. Small Rumin Res.

[12] Huang J, Sun Y (2012). Development of a loop-mediated isothermal amplification method for rapid detection of caprine arthritis-encephalitis virus proviral DNA. Arch Virol.

[13] Kaba J, Strzałkowska N (2012). Twelve-year cohort study on the influence of caprine arthritis–encephalitis virus infection on milk yield and composition. J Dairy Sci.

[14] Konishi M, Tsuduku S (2004). An epidemic of caprine arthritis encephalitis in Japan: isolation of the virus. J Vet Med Sci.

[15] Kuzmak J, Rola M (2007). Molecular characterization of lentiviruses from goats from Poland based on gag gene sequence analysis. Comp Immunol Microbiol Infect Dis.

[16] Leroux C, Lerondelle C (1997). RT-PCR detection of lentiviruses in milk or mammary secretions of sheep or goats from infected flocks. Vet Res.

[17] Luengo C, Sanchez A (2004). Influence of intramammary infection and non-infection factors on somatic cell counts in dairy goats. J Dairy Res.

[18] Martins G, Penna B (2012). Leptospirosis as the most frequent infectious disease impairing productivity in small ruminants in Rio de Janeiro, Brazil. Trop Anim Health Prod.

[19] Modolo JR, Stachissini VM (2009). PCR associated with agar gel immunodiffusion assay improve caprine arthritis–encephalitis (CAEV) control. Small Rumin Res.

[20] Narayan O, Cork LC (1985). Lentiviral diseases of sheep and goats: chronic pneumonia leukoencephalomyelitis and arthritis. Rev Infect Dis.

[21] Narayan O, Kennedy-Stoskopf S (1983). Activation of caprine arthritis–encephalitis virus expression during maturation of monocytes to macrophages. Infect Immun.

[22] Nord K, Rimstad E (1998). Prevalence of antibodies against caprine arthritis–encephalitis virus in goat herds in Norway. Small Rumin Res.

[23] Oem JK, Chung JY et al (2012) Large-scale serological survey of caprine arthritis–encephalitis virus (CAEV) in Korean Black Goats (Capra hircus aegagrus). J Vet Med Sci 74(12):1657–1659 10.1292/jvms.12-0103 22814087

[24] Peterhans E, Greenland T, Badiola J et al (2004) Routes of transmission and consequences of small ruminant lentiviruses (SRLVs) infection and eradication schemes. Vet Res 35:257–274 10.1051/vetres:2004014 15210075

[25] Ponti W, Paape M (2008). Phenotypic alteration of blood and milk leukocytes in goats naturally infected with caprine arthritis-encephalitis virus (CAEV). Small Rumin Res.

[26] Qu J, Liu H (2005). Current situation of research on caprine arthritis encephalitis. Chin J Prev Med.

[27] Rosati S, Pittau M (1995). Genetic and antigenic characterization of caev (caprine arthritis–encephalitis virus) recombinant transmembrane protein. Vet Microbiol.

[28] Rosati S, Profiti M (2004). Development of recombinant capsid antigen/transmembrane epitope fusion proteins for serological diagnosis of animal lentivirus infections. J Virol Methods.

[29] Rowe JD, East NE (1997). Risk factors for transmission and methods for control of caprine arthritis–encephalitis virus infection. Vet Clin North Am Food Anim Pract.

[30] Rowe JD, East NE (1992). Risk factors associated with the incidence of seroconversion to caprine arthritis-encephalitis virus in goats on California dairies. Am J Vet Res.

[31] Sanchez A, Contreras A (2001). Relationships between infection with caprine arthritis encephalitis virus, intramammary bacterial infection and somatic cell counts in dairy goats. Vet Rec.

[32] Synge BA, Ritchie CM (2010). Elimination of small ruminant lentivirus infection from sheep flocks and goat herds aided by health schemes in Great Britain. Vet Rec.

[33] Torres-Acosta JFJ, Gutierrez-Ruiz EJ (2003). Serological survey of caprine arthritis-encephalitis virus in 83 goat herds of Yucatan, Mexico. Small Rumin Res.

[34] Turin L, Pisoni G (2005). Correlation between milk parameters in CAEV seropositive and negative primiparous goats during an eradication program in Italian farm. Small Rumin Res.

[35] Zanoni RG, Nauta IM (1991). Expression in Escherichia coli and sequencing of the coding region for the capsid protein of Dutch maedi-visna virus strain ZZV 1050: application of recombinant protein in enzyme-linked immunosorbent assay for the detection of caprine and ovine lentiviruses. J Clin Microbiol.

